# Timing of antiretroviral therapy initiation affects intact HIV reservoirs following analytical treatment interruption

**DOI:** 10.1172/JCI181632

**Published:** 2024-10-15

**Authors:** Maegan R. Manning, Jana Blazkova, Jesse S. Justement, Victoria Shi, Brooke D. Kennedy, M. Ali Rai, Catherine A. Seamon, Kathleen Gittens, Michael C. Sneller, Susan Moir, Tae-Wook Chun

**Affiliations:** 1 Laboratory of Immunoregulation, National Institute of Allergy and Infectious Diseases and; 2 Critical Care Medicine Department, Clinical Center, NIH, Bethesda, Maryland, USA.

**Keywords:** AIDS/HIV, Drug therapy

**To the Editor:** While current antiretroviral therapy (ART) has proven to be effective in suppressing HIV, the eradication of the virus remains elusive in people living with HIV (PLWH). Given that HIV suppression in PLWH requires lifelong adherence to ART, there is a need to investigate novel therapies aimed at achieving ART-free virologic remission following analytical treatment interruption (ATI) (1). It has been shown that ATI results in the transient expansion of HIV reservoirs in PLWH during the viremic phase (2); however, the detailed dynamics of the intact proviral HIV DNA (IPD) reservoir following reinitiation of ART in PLWH who initiated ART during the acute/early (A/E) versus chronic phase of infection have not been fully delineated. We conducted the present study to address this issue.

We studied two cohorts of PLWH: those who initiated ART during the A/E (ClinicalTrials.gov NCT01859325) (3) or chronic (ClinicalTrials.gov NCT03225118) (4) phase of infection (Supplemental Table 1; supplemental material available online with this article; https://doi.org/10.1172/JCI181632DS1). All blood products were obtained in accordance with protocols approved by the Institutional Review Board of the National Institute of Allergy and Infectious Diseases, NIH. All study participants provided written, informed consent. We studied the following time points: before ATI (pre-ATI), during ATI (ATI), and 24 and 52 or more weeks after reinitiation of ART (post-ATI1 and post-ATI2, respectively). As shown in Figure 1A, both groups experienced a significant increase in the level of IPD during ATI. After the reinitiation of ART, the IPD burden in the A/E group normalized to the pre-ATI level by post-ATI1. However, in the chronic group, the level of IPD remained elevated at post-ATI1 and post-ATI2 (*P* = 0.0040 and *P* = 0.0046, respectively) compared with that of pre-ATI. Both 5′ and 3′ defective HIV DNA levels returned to pre-ATI levels by post-ATI1 for both groups. We found no correlation between the IPD fold change (post-ATI1/pre-ATI) and the pre-ATI IPD when all study participants were included (*P* = 0.0694), but a significant correlation for the chronic group alone (*P* = 0.0138) (Figure 1B). There was also no correlation between the IPD fold change and the plasma viremia before ART reinitiation nor at the peak (Figure 1C), suggesting that the time to normalize the IPD was not driven by plasma viremia during ATI. Next, we measured residual plasma viremia (<40 copies/ml) at pre-ATI and post-ATI1 for both cohorts (Figure 1D). The level of residual plasma viremia was significantly higher at post-ATI1 compared with pre-ATI (*P* = 0.0181) for the chronic group, but there was no difference for the A/E group. We then conducted longitudinal analyses of immune markers to address the role of host immunity in the normalization of the HIV reservoirs in the study populations. Among the biomarkers examined, we found that levels of soluble programmed death-ligand 1 (PD-L1) (*P* = 0.0066) and perforin (*P* = 0.0094) remained significantly elevated at post-ATI1 in the chronic group but not in the A/E group (Figure 1E and Supplemental Figure 1A). We also conducted high-dimensional immune profiling of peripheral blood T cells of study participants at pre-ATI, ATI, and post-ATI1 (Figure 1F and Supplemental Figure 1B). Of the 15 metaclusters generated by the FlowSOM algorithm, cluster 11, defined by markers corresponding to mucosal-associated invariant T (MAIT) cells (5), showed a significantly higher frequency in A/E compared with the chronic group at pre-ATI, ATI, and post-ATI1 time points (*P* = 0.0108, *P* = 0.0180, *P* = 0.0229, respectively). Conversely, we found no differences in the levels of HIV-specific T cells between the two cohorts at post-ATI1 (Figure 1G). Finally, phenotypic analyses of NK cells at the post-ATI1 time point revealed significantly higher levels of cytotoxic effector cells (CD56^dim^CD16^+^CD57^+^) (6) in the A/E group compared with the chronic group (*P* = 0.0131), suggesting a potential role of NK cells in modulating HIV reservoirs after ART reinitiation (Figure 1H).

Our study highlights the impact of the timing of ART initiation on the dynamics of the IPD reservoir in PLWH who reinitiated ART following ATI. Our data suggest that the kinetics of plasma viremia during ATI is not a major determining factor for the normalization of the IPD reservoir following reinitiation of ART. Rather, a larger reservoir size at baseline and the persistence of residual plasma viremia and immune activation/inflammation at the post-ATI1 time point in the chronic group may have collectively contributed toward delayed normalization of the IPD reservoir. Although the levels of HIV-specific T cells were comparable between the two groups following reinitiation of ART, certain immune cells, such as MAIT cells and a subset of highly cytotoxic NK cells, may have played a key role in facilitating the normalization of the IPD reservoirs in the A/E group. Major caveats of our work include the following: (a) the small sample size, (b) the inclusion of PLWH who participated in separate ATI trials, (c) the lack of sequencing information, and (d) the clinical relevance of our work. Nonetheless, our findings help delineate the underlying immunologic and virologic mechanisms that may explain differences in the normalization of the IPD reservoir in the A/E versus the chronic group. This insight could potentially lead to the development of novel therapies and aid in the interpretation of results from clinical trials involving ATI in PLWH.

For further information, see Supplemental Methods.

## Data availability.

Values for all data points in graphs are reported in the Supporting Data Values file.

## Supplementary Material

Supplemental data

Supporting data values

## Figures and Tables

**Figure 1:**
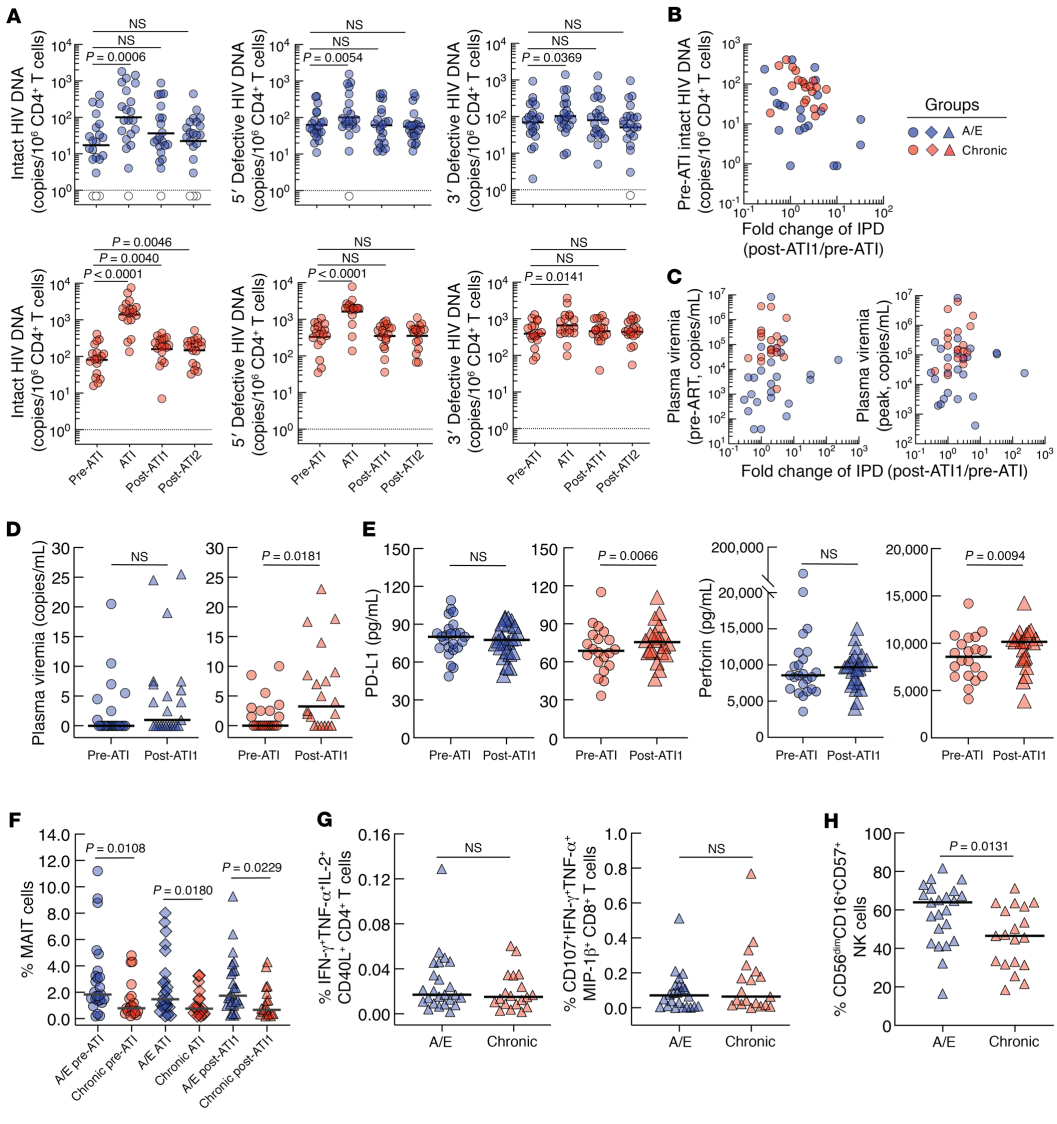
Immunologic and virologic parameters. Intact and defective HIV proviral DNA (**A**), correlations (Spearman’s method) between IPD fold change (post-ATI1/pre-ATI) and pre-ATI IPD (**B**) or plasma viremia during ATI (**C**), pre-ATI and post-ATI1 residual plasma viremia (**D**), pre-ATI and post-ATI1 levels of PD-L1 and perforin (**E**), frequency of MAIT cells (**F**), polyfunctional HIV-specific T cell response (**G**), and percentage of CD56^dim^CD16^+^CD57^+^ NK cells (**H**). Open symbols indicate values under the limit of detection, and black bars indicate the geometric mean or median. *P* values were determined using Wilcoxon’s matched-pairs signed rank test (**A**, **D**, and **E**) and adjusted using the Holm-Bonferroni method (**A**) and the Mann-Whitney *U* test (**F**–**H**).

## Version 1 10/15/2024

Electronic publication

## References

[1] Landovitz RJ (2023). Prevention, treatment and cure of HIV infection. Nat Rev Microbiol.

[2] Wen Y (2018). Lessons learned from HIV antiretroviral treatment interruption trials. Curr Opin HIV AIDS.

[3] Sneller MC (2017). A randomized controlled safety/efficacy trial of therapeutic vaccination in HIV-infected individuals who initiated antiretroviral therapy early in infection. Sci Transl Med.

[4] Sneller MC (2020). Kinetics of plasma HIV rebound in the era of modern antiretroviral therapy. J Infect Dis.

[5] Sandberg JK (2023). The emerging role of MAIT cell responses in viral infections. J Immunol.

[6] Blazkova J (2023). Correlation between TIGIT expression on CD8+ T cells and higher cytotoxic capacity. J Infect Dis.

