# Supplementary material for: Timing of antiretroviral therapy initiation affects intact HIV reservoirs following analytical treatment interruption
Source: J Clin Invest. 2024 Oct 15;134(20):e181632. doi: 10.1172/JCI181632 (PMC11473155; doi:10.1172/JCI181632)
Supplement: Supplemental data [file jci-134-181632-s056.pdf]

Supplemental Materials

Supplemental Methods

Sex as a biological variable

Our study examined male and female study participants living with HIV.

Study participants

Our study consisted of 24 PLWH who initiated ART during the acute/early phase of infection (A/E, ClinicalTrials.gov ID NCT01859325) and 19 PLWH who initiated ART during the chronic phase of infection (Chronic, ClinicalTrials.gov ID NCT03225118). The Post-ATI2 timepoint had a median of 52 weeks for the Chronic group and 92 weeks (range, 48-153) for the A/E group. All study participants underwent ATI and blood and leukapheresis products were obtained under clinical protocols approved by the Institutional Review Boards of the National Institute of Allergy and Infectious Diseases at the National Institutes of Health. The ART reinitiation criteria of the A/E group are as follows: 1) a confirmed >30% decline in baseline CD4 cell count or an absolute CD4 cell count <350 cells/mm^3, 2) a sustained (≥4 weeks) HIV RNA level of >50,000 copies/ml, and 3) any HIV-related symptoms. The ART reinitiation criteria of the Chronic group are as follows: 1) a confirmed >30% decline in baseline CD4 cell count or an absolute CD4 cell count <350 cells/mm^3, 2) a sustained (≥3 weeks) HIV RNA level of >1,000 copies/ml or one measurement of HIV RNA level >50,000 copies/ml, 3) any HIV-related symptoms or acute retroviral syndrome presenting as fever, lymphadenopathy, sore throat, rash, myalgia/arthralgia, diarrhea not already determined by physician examination to be related to something other than HIV, 4) any development of HIV specific opportunistic infections, 5) at month 6, any subject who has not met criteria to end the ATI but has detectable viremia (>40 copies/ml) will be instructed to

restart ART, and 6) pregnancy. The inclusion and exclusion criteria of the original clinical trials are as follows:

A/E group inclusion criteria

1. Age, 18-65 years.
2. Institution of ART within 12 weeks of being diagnosed with acute or early HIV-1 infection.

Acute HIV-1 infection is defined as:

a. Detectable plasma HIV-1 RNA levels of >2000 copies/mL with a negative result from an HIV-1 EIA, or

b. Positive result from an HIV-1 EIA with a negative or indeterminate result from an HIV-1 western blot that subsequently evolves to a confirmed positive result, or

c. Negative result from an HIV-1 EIA within the past 4 months and HIV-1 RNA levels of >400,000 copies/mL, in the setting of a potential exposure to HIV-1.

Early HIV-1 infection is defined as:

a. Negative result from an HIV-1 EIA within 6 months prior to a positive result from an HIV-1 EIA and an HIV-1 western blot.
b. Negative result from a rapid HIV-1 test within 1 month prior to a positive result from an HIV-1 EIA and an HIV-1 western blot.
c. Presence of low level of HIV antibodies as determined by having a positive EIA or a positive Western blot with a non-reactive detuned EIA according to a serologic testing algorithm for recent infection.

3. $CD4^+$ cell count >450 cells/mm^3 at screening.

4. Documentation of continuous ART treatment with suppression of plasma viral level below the limit of detection for >1 year. Subjects with a single "blip" (i.e., detectable viral levels on ART) prior to randomization may be included provided they satisfy the following criteria:
    a. The blips are <400 copies/mL, and
    b. Succeeding viral levels return to levels below the limit of detection on subsequent testing.
5. Willingness to undergo ATI.
6. Laboratory values within pre-defined limits at screening:
    a. Absolute neutrophil count >1,000/mm^3.
    b. Hemoglobin levels >10.0 g/dL for men and >9.0 g/dL for women.
    c. Platelet count >100,000/mm^3.
    d. Prothrombin time (PT) and partial thromboplastin time (PTT) <1.5 upper limit of normal (ULN).
    e. Estimated or a measured creatinine clearance rate of >60 mL/min as determined by the NIH Clinical Center laboratory.
    f. AST and ALT levels of <2.5 x ULN.
7. Willingness to have samples stored for future research.
8. Women of childbearing potential must have a negative pregnancy test result and must agree to use an adequate form of contraception.

<u>A/E group exclusion criteria</u>

1. Allergy to amide-type local anesthetics (bupivacaine [Marcaine], lidocaine [Xylocaine], Mepivacaine [Polocaine/Carbocaine], etidocaine [Duranest], prilocaine [Citanest, EMLA cream]).

2. Chronic hepatitis B, as evidenced by a positive test for hepatitis B surface antigen (HBsAg), or chronic hepatitis C virus (HCV) infection, as evidenced by a positive test for HCV RNA. Subjects with a positive test for HCV antibody and a negative test for HCV RNA are eligible.
3. Changes in cART regimen due to virologic breakthrough.
4. HIV immunotherapy or vaccine(s) received within 1 year prior to screening.
5. Any licensed or experimental non-HIV vaccination (e.g., hepatitis B, influenza, pneumococcal polysaccharide) received within 4 weeks prior to study entry.
6. Interruption of cART for >3 months since its initiation.
7. Pregnancy or planned pregnancy during the study period or breastfeeding.
8. Any active malignancy that may require systemic chemotherapy or radiation therapy.
9. Immunosuppressive medications received within 6 months before the first study vaccination (Not excluded: [1] corticosteroid nasal spray for allergic rhinitis; [2] topical corticosteroids for mild, uncomplicated dermatitis; or [3] oral/parenteral corticosteroids administered for non-chronic conditions not expected to recur [length of therapy ≤10 days, with completion in ≥30 days prior to enrollment]).
10. Evidence of hepatic decompensation in subjects with cirrhosis: history of ascites, hepatic encephalopathy, or bleeding esophageal varices, or screening laboratory results with any of the following:
    a. International normalized ratio of ≥1.5 x ULN.
    b. Serum albumin <3.2 g/dL.
    c. Serum total bilirubin >1.8 x ULN, unless history of Gilbert's disease or deemed related to treatment with atazanavir.
11. History or other clinical evidence of:

a. Significant or unstable cardiac disease (e.g., angina, congestive heart failure, recent myocardial infarction, significant arrhythmia).
b. Severe illness, malignancy, immunodeficiency other than HIV, or any other conditions that, in the opinion of the investigator, would make the subject unsuitable for the study.
c. AIDS-defining condition.

12. Known allergy or sensitivity to the components of the investigational therapy.
13. History of significant cardiac arrhythmia (e.g., supraventricular tachycardia, ventricular tachycardia, and atrial fibrillation/flutter).
14. Active drug or alcohol use or any other pattern of behavior that, in the opinion of the investigator, would interfere with adherence to study requirements.
15. Any active systemic inflammatory or autoimmune disease or condition.
16. Presence of implanted electronic medical device (e.g., pacemaker, implantable cardiac defibrillator) or surgical/traumatic metal implant in the upper limb and/or upper torso.
17. Neurological or neuropsychiatric disorder that may interfere with the assessment of safety (e.g., frequent recurring headaches, for example, a pattern of >1 headache/month affecting activities of daily living/work, frequent or severe/complicated migraines, cluster headaches); or history of encephalitis, narcolepsy, stroke with sequelae, moderate/severe major depressive disorder, moderate/severe bipolar disorder, seizure disorder.
18. Deltoid skinfold measurements (by caliper) of >40 mm.
19. Body mass index >40.

<u>Chronic group inclusion criteria</u>

1. Age, 18-65 years
2. Documented HIV-1 infection and clinically stable

3. In general good health, with an identified primary health care provider for medical management of HIV infection and willing to maintain a relationship with a primary health care provider for medical management of HIV infection while participating in the study
4. $CD4^+$ T cell count >450 cells/mm^3 at screening
5. Documentation of continuous ART treatment with suppression of plasma viral level below the limit of detection for ≥3 years. Subjects with "blips" (i.e., detectable viral levels on ART) prior to screening may be included provided they satisfy the following criteria:
   a. The "blips" are <400 copies/mL, and
   b. Succeeding viral levels return to levels below the limit of detection on subsequent testing
6. Willingness to undergo ATI
7. Willingness to restart ART once restart criteria are met
8. Willingness for both male and female participants to agree to use barrier protection methods during the ATI to decrease the risk of HIV transmission
9. Willingness of both male and female participants of childbearing potential must agree to use adequate contraception prior to study entry and for the duration of study participation.
10. Laboratory values within pre-defined limits at screening:
   a. Absolute neutrophil count >1,000/mm^3
   b. Hemoglobin (Hgb) levels >10.0 g/dL for men and >9.0 g/dL for women
   c. Platelet count >150,000/mm^3
   d. Estimated glomerular filtration rate (eGFR) ≥60 mL/min as determined by the NIH Clinical Center (CC) laboratory
   e. Aspartate aminotransferase (AST) and alanine aminotransferase (ALT) levels of <2.5 x upper limit normal (ULN)

11. Willingness to have samples stored for future research

Chronic group exclusion criteria

1. Chronic hepatitis B, as evidenced by a positive test for hepatitis B surface antigen (HBsAg), an isolated positive Hepatitis B core antibody (negative HBsAg and anti-HBV Ab) and/or positive Hepatitis B virus (HBV) DNA.
2. Chronic hepatitis C virus (HCV) infection as evidenced by a positive test for HCV RNA.
3. Has a history of institution of ART within 12 weeks of being diagnosed with acute or early HIV-1, where acute/early infection is defined by any one of the following:
   a. Positive HIV-1 enzyme immunoassay (EIA) with negative/indeterminate HIV-1 western blot that subsequently becomes positive

   b. Negative HIV-1 EIA within the past 4 months and HIV-1 RNA levels of >400,000 copies/mL, in the setting of a potential exposure to HIV-1.

   c. Negative rapid HIV-1 within one month prior to a positive HIV-1 EIA and HIV-1 western blot

   d. Low level HIV antibodies (positive EIA or western blot) with a non-reactive detuned EIA
4. Documented nadir $CD4^+$ T cell count <200 cells /mm^3
5. Any history of opportunistic infections
6. Subjects with history of receiving ART consisting of mono or dual drug therapy
7. Documented multiclass antiretroviral drug resistance that, in the judgement of the investigator, would pose a risk of virologic failure should additional mutations develop during the study

8. Any experimental non-HIV vaccination received within 2 weeks prior to study enrollment and at any time during the study.
9. Any licensed vaccine (e.g., hepatitis B, influenza, pneumococcal polysaccharide) received within 2 weeks prior to the study enrollment at the time of signing consent
10. Receipt of other investigational study agent within 28 days of enrollment and at any time during the study
11. Any active malignancy that may require systemic chemotherapy or radiation therapy
12. Systemic immunosuppressive medications received within 3 months prior to enrollment. The following are not excluded: [1] corticosteroid nasal spray or inhaler; [2] topical corticosteroids for mild, uncomplicated dermatitis; and [3] oral/parenteral corticosteroids administered for non-chronic conditions not expected to recur (length of therapy ≤10 days, with completion in ≥30 days prior to enrollment)
13. History of or other clinical evidence of:
    a. Significant or unstable cardiac disease (e.g., angina, congestive heart failure, recent myocardial infarction)
    b. Severe illness, malignancy, immunodeficiency other than HIV, active systemic infection other than HIV, or any other condition that, in the opinion of the investigator, would make the subject unsuitable for the study
14. Active drug or alcohol abuse or any other pattern of behavior that, in the opinion of the investigator, would interfere with adherence to study requirements

Intact HIV Proviral DNA Assay (IPDA)

Intact and defective HIV DNA were quantified by the intact proviral DNA assay (IPDA) using digital PCR (QIAGEN) and modified primers and probes as previously described (1-3).

Highly enriched $CD4^+$ T cells were used to isolate genomic DNA with the QIAamp DNA Mini Kit (QIAGEN). HIV-specific and RPP30-specific primers and probes were used for amplification. The following primers and probes were used for HIV Gag: 5’-GACTAGCGGAGGCTAGAAGGAGAGA-3’ (5’ primer), 5’-CTAATTCTCCCCCGCTTAATAYTGACG-3’ (3’ primer), and 5’-6FAM-A+T+GGG+TG+CGAGA-IABkFQ-3’ (LNA probe). The following primers and probes were used for HIV Env: 5’- AGTGGTGCAGAGAGAAAAAAGAGC-3’ (5’ primer), 5’-GTCTGGCCTGTACCGTCAGC-3’ (3’ primer), 5’-VIC- CCTTGGGTTCTTGGGA-MGB-3’ (probe), and 5’- CCTTAGGTTCTTAGGAGC-MGB-3’ (unlabeled hypermutated probe). The following primers and probes were used for housekeeping gene RPP30: 5’-GATTTGGACCTGCGAGCG-3’ (RPP30-1 5’ primer), 5’- GCGGCTGTCTCCACAAGT-3’ (RPP30-1 3’ primer), 5’-6FAM-TTCTGACCTGAAGGCTCTGCGC-IABkFQ-3’ (RPP30-1 probe), 5’- GTGTGAGTCAATCACTAGACAGAA-3’ (RPP30-2 5’ primer), 5’-AAACTGCAACAACATCATAGAGC-3’ (RPP30-2 3’ primer), and 5’-HEX-AGAGAGCAACTTCTTCAAGGGCCC-IABkFQ-3’ (RPP30-2 probe). Each PCR reaction was done in triplicate. The copy numbers of intact and defective HIV proviral DNA were normalized per $1x10^6$ $CD4^+$ T cells and adjusted using the DNA shearing index (DSI).

Measurements of T Cell and NK Phenotypes

Peripheral blood T cells were stained with Zombie NIR (Biolegend #423106), fluorophore-conjugated antibodies, and Brilliant Stain Buffer Plus (BD #566385). Antibodies used for T cell phenotyping included: CD3-BUV805 (clone SK7, BD #612893), CD4-BUV395 (clone RPA-T4, BD #564724), CD8-BUV737 (clone SK1, BD #612754), CD45RA-PerCP-Cy5.5 (clone HI100, BD #563429), CCR7-V450 (clone 150503, BD #560863), CXCR4-BV510

(clone 12G5, Biolegend #306536), CCR4-BUV615 (clone 1G1, BD #613000), CCR5-BV650 (clone 3A9, BD #564999), CXCR3-BV711 (clone G025H7, Biolegend #353732), CCR6-APC/Fire750 (clone G034E3, Biolegend #353443), CD27-BV421 (clone O323, Biolegend #302824), CD28-BV480 (clone CD28.2, BD #566110), CD127-BUV563 (clone eBioRDR5, eBioscience #365-1278-42), CD38-APC/Fire810 (clone HB-7, Biolegend #356644), CD226-BUV496 (clone DX11, BD #749935), CD69-BV605 (clone FN50, Biolegend #310938), CD25-BV785 (clone BC96, Biolegend #302638), CD95-PerCP-eFluor 710 (clone DX2, eBioscience #46-0959-42), KLRG1-APC (clone 2F1/KLRG1, Biolegend #138412), PD-1-PE-Cy7 (clone EH12.1, BD #561272), CD160-Alexa Fluor 647 (clone BY55, BD #562362), CD161-PE-Cy5 (clone HP-3G10, Biolegend #339951), CD62L-R718 (clone DREG-56, BD #567988), HLA-DR-Super Bright 436 (clone LN3, eBioscience #62-9956-42), TIGIT-PE-eFluor 610 (clone MBSA43, eBioscience #61-9500-42), 2B4-PE (clone C1.7, eBioscience #12-5838-42), CD45-SparkBlue550 (clone 2D1, Biolegend #368550), and CD57-BB515 (clone NK-1, BD #565285). Antibodies used for NK cell phenotyping included: CD3-BUV805 (clone SK7, BD #612893), CD16-BUV737 (clone 3G8, BD #612786), CD56-FITC (clone HCD56, Biolegend #318304), CD57-BV421 (clone NK-1, BD #568894), NKp30-APC (clone P30-15, Biolegend #325210), NKG2D-PerCP-Cy5.5 (clone 1D11, Biolegend #320818), CD7-PE-Cy7 (clone CD7-6B7, Biolegend #343114), CD161-Alexa Fluor 700 (clone HP-3G10, Biolegend #339942), CD11b-Pacific Blue (clone ICRF44, Biolegend #301315), CD19-BV650 (clone SJ25C1, BD #563226), and Siglec-7-PE (clone 6-434, Biolegend #339204). Data were collected on a spectral flow cytometer Cytek Aurora using the SpectroFlo Software (Cytek Biosciences) and analyzed using FlowJo version 10.7.1 and the OMIQ platform (www.Omiq.ai).

High-dimensional flow cytometry

Uniform Manifold Approximation and Projection (UMAP) dimensionality reduction and FlowSOM clustering algorithms were conducted using the OMIQ platform (Omiq.ai) to analyze flow cytometry data. An equal sampling of approximately 100,000 $CD3^+$ T cells from each FCS data file was used for the above analysis. The UMAP maps were generated based on the expression of the following markers: CD4, CD8, CD45RA, CCR7, CD62L, CD27, CD28, CD38, HLA-DR, CD226, TIGIT, PD-1, 2B4, CD160, KLRG1, CD57, CXCR3, CCR4, CCR6, CD161, CD127, CD25, CD69, CD95, CXCR4, and CCR5. Using hierarchical consensus clustering, the self-organizing map (SOM) was generated and 15 meta-clusters were identified. A heatmap displaying column-scaled z-scores of mean fluorescent intensity for each cluster was generated using the OMIQ platform (Omiq.ai).

Examination of Biomarkers in plasma

Levels of plasma-associated biomarkers were determined using the ELLA (ProteinSimple) platform as instructed by the manufacturer. The biomarkers measured were B7-H1, CXCL10, IL-ra2, IL-6, Perforin, and E-Cadherin.

Measurements of Residual Plasma Viremia

Levels of residual plasma viremia (<20 copies/ml) were determined in quadruplicate as previously described (4) using the COBAS 5800 system per the manufacturer's instructions.

Measurements of HIV-specific immune response

The frequency of HIV Gag-specific $CD4^+$ and $CD8^+$ T cells in each study participant was determined by intracellular cytokine staining. PBMCs were incubated for 6 hours at 37°C with overlapping HIV Gag peptides (HIV Reagent Program), brefeldin A (Sigma-Aldrich), and anti-CD107a-BUV510 antibody (clone H4A3, BD #563078). Cells were then stained with Zombie

NIR (Biolegend #423106) and the following antibodies for surface markers: CD3-BUV805 (clone SK7, BD #612893), CD4-BUV395 (clone RPA-T4, BD #564724), and CD8-BUV737 (clone SK1, BD #612754). Subsequently, cells were fixed (BD #347692) and permeabilized (BD #349202) before incubation with intracellular antibodies: Brilliant Stain Buffer Plus (BD #566385), IFN-γ-APC (clone B27, BD #554702), TNF-α-BV650 (clone MAb11, BD #563418, CD40L-BV421 (clone TRAP1, BD #563886), IL-2- PerCP-eF710 (clone MQ1-17H12, eBioscience #46-7029-42) and MIP-1ß-PE (clone D21-1351, BD #550078). Data were collected on a spectral flow cytometer Cytek Aurora using the SpectroFlo Software (Cytek Biosciences) and analyzed using FlowJo version 10.7.1.

Statistical analysis

P values were determined using the Wilcoxon matched-pairs signed rank test (Figure 1A, D, and E) and adjusted using the Holm-Bonferroni method (Figure 1A) and the two-tailed Mann-Whitney test (Figure 1F-H and Supplemental Figure 1A). Correlations were determined by the Spearman method (Figure B-C). All tests were performed with Prism 10 (GraphPad).

Study approval

All blood products were obtained in accordance with protocols approved by the Institutional Review Board of the National Institute of Allergy and Infectious Disease, National Institutes of Health, Bethesda, Maryland. All study participants provided written informed consent.

Data availability

A single XLS file XLS containing values for all graphs shown in the manuscript is available and reported in the Supporting Data file.

Supplemental Table 1. Clinical characteristics of study participants.

	**Acute/Early (N=24)**	**Chronic (N=19)**	***P* values**
**Sex, no. (%)**			
Male	24 (100)	17 (89)	
Female	0	2 (11)	
**Age**			
Mean	42	51	
(Range)	(21-65)	(38-61)	***P* = 0.0009**
**$CD4^+$ T Cell Count (cells/mm^3) Prior to ATI**			
Median	738	724	
(Range)	(363-2162)	(466-1778)	*P* = 0.5569
**$CD4^+$ T Cell % (cells/mm^3) Prior to ATI**			
Median	39	42	
(Range)	(29-62)	(29-51)	*P* = 0.1984
**$CD8^+$ T Cell Count (cells/mm^3) Prior to ATI**			
Median	522	648	
(Range)	(307-1600)	(296-1587)	*P* = 0.2715
**$CD8^+$ T Cell % (cells/mm^3) Prior to ATI**			
Median	31	34	
(Range)	(14-53)	(21-52)	*P* = 0.2258
**Duration of ART (years)**			
Median	3	8	
(Range)	(1-19)	(2-16)	***P* = 0.0318**
**Duration of ATI (days)**			
Median	121	41	
(Range)	(47-319)	(16-109)	***P* < 0.0001**
**Duration of HIV Infection prior to Initiation of ART (months)**			
Median (Range)	1.3 (0-155)	68.3 (0-329)	***P* < 0.0001**
**Duration of HIV Suppression prior to ATI (years)**			
Median	3.6	7.6	
(Range)	(2-20)	(2-16)	*P* = 0.4708
**Time to Viral Rebound During ATI (days)**			
Median	28	13	
(Range)	(11-70)	(7-38)	***P* = 0.0004**
**Time to Reinitiate ART (days)**			
Median	121	35	***P* < 0.0001**
(Range)	(47-319)	(8-107)	
**Time to Viral Resuppression after ART Reinitiation (days)**			
Median			
(Range)	30	55	
	(13-172)	(5-107)	*P* = 0.5520
**CD4 Nadir**			
Median	514	290	
(Range)	(210-1348)	(163-800)	***P* < 0.0001**

**Peak Plasma Viremia During ATI (copies/mL)**<br>Median<br>(Range)	 28,703<br>(416-8405097)	 151,332<br>(20922-6315485)	 <br>***P* = 0.0018**
**Pre-ATI Intact Proviral DNA (copies/10^6 $CD4^+$ T cells)**<br>Median<br>(Range)	 21<br>(0-409)	 86<br>(16-407)	 <br>***P* = 0.0029**
**Pre-ATI Plasma Viremia (copies/mL)**<br>Median<br>(Range)	 <40<br>(<40)	 <40<br>(<40)	

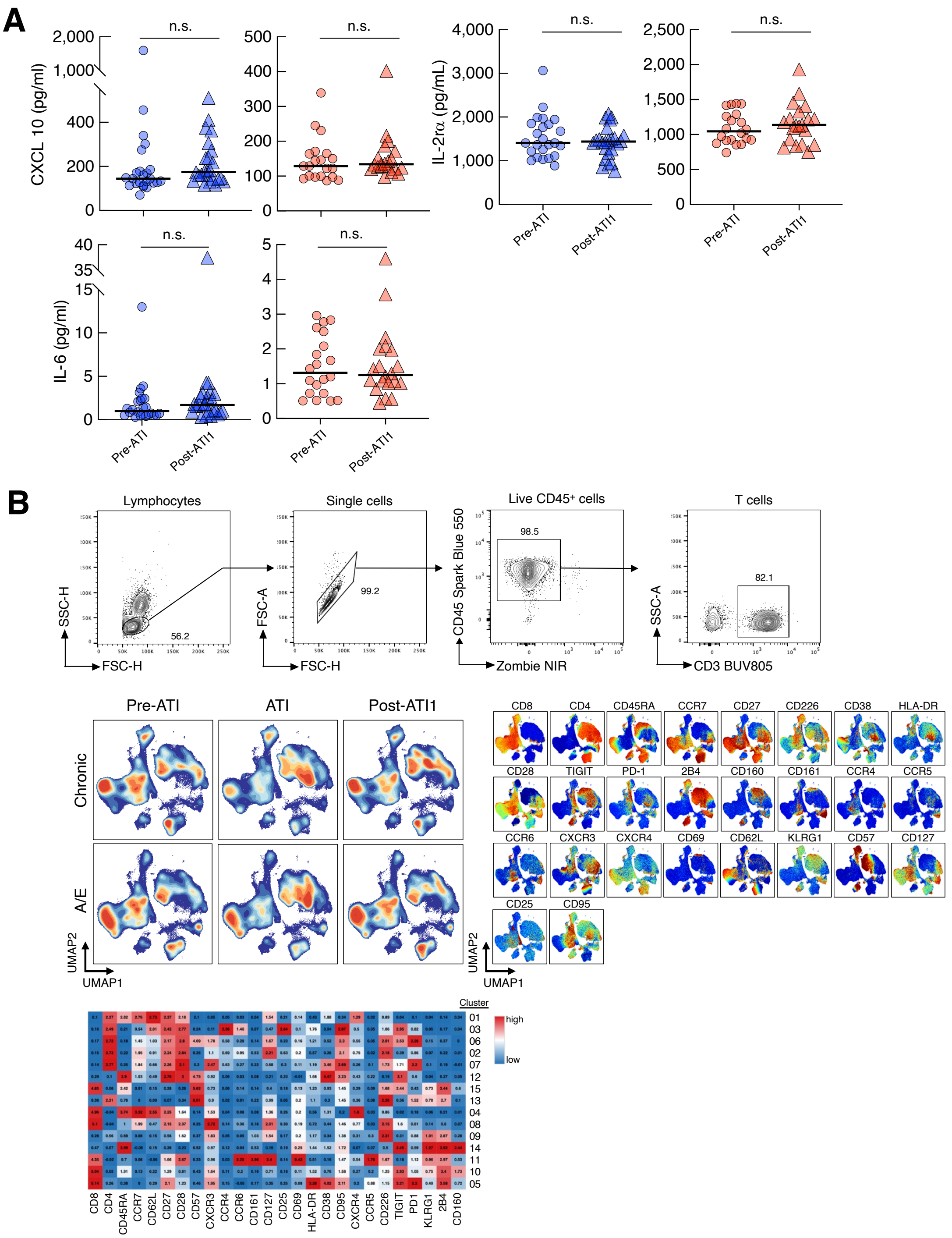

Supplemental Figure 1: Levels of biomarkers in plasma and T cell immune parameters in the A/E and Chronic group. (A) Pre-ATI and Post-ATI1 levels of CXCL10, IL-2Rα, and IL-6. (B) T cell immune parameters in the A/E and Chronic group. Gating strategy (top), uniform manifold approximation and projection (UMAP) plots of $CD3^+$ T cells from the A/E and Chronic group Pre-ATI and Post- ATI1 (middle left) and UMAP visualization of expression of the indicated markers (middle right). A heatmap showing the level of expression of individual markers in each cluster (bottom).

Acknowledgements

We thank the volunteers for their participation in this study. This work was supported by the Intramural Research Program of the NIAID, NIH.

Author contributions

MRM and TWC designed the research. MRM, JB, JSJ, VS, BDK, and TWC performed the research. CAS, KG, and MCS contributed research materials. MRM, JB, SM, and TWC analyzed the data. MRM, SM, and TWC wrote the manuscript. All authors reviewed and approved the manuscript.

Supplemental References

1. Bruner KM, Wang Z, Simonetti FR, Bender AM, Kwon KJ, Sengupta S, et al. A quantitative approach for measuring the reservoir of latent HIV-1 proviruses. *Nature.* 2019;566(7742):120-5.
2. Levy CN, Hughes SM, Roychoudhury P, Reeves DB, Amstuz C, Zhu H, et al. A highly multiplexed droplet digital PCR assay to measure the intact HIV-1 proviral reservoir. *Cell Rep Med.* 2021;2(4):100243.
3. Blazkova J, Whitehead EJ, Schneck R, Shi V, Justement JS, Rai MA, et al. Immunologic and virologic parameters associated with HIV DNA reservoir size in people living with HIV receiving antiretroviral therapy. *J Infect Dis.* 2023.
4. Chun TW, Murray D, Justement JS, Hallahan CW, Moir S, Kovacs C, and Fauci AS. Relationship between residual plasma viremia and the size of HIV proviral DNA

reservoirs in infected individuals receiving effective antiretroviral therapy. *J Infect Dis.* 2011;204(1):135-8.
